# Exploring the perceptions and lived experiences of family members living with people diagnosed with COVID-19 in South Africa: a descriptive phenomenological study

**DOI:** 10.1080/17482631.2023.2247622

**Published:** 2023-08-28

**Authors:** Celenkosini Thembelenkosini Nxumalo, Lwandile Tokwe, Silingene Joyce Ngcobo, Nkululeko Phalson Gam, Gugu Gladness Mchunu, Lufuno Makhado

**Affiliations:** a Academic Development Unit, Faculty of Health Sciences, Durban University of Technology, Ritson Campus, Durban, South Africa; b Department of Nursing, School of Nursing and Public Health, University of KwaZulu-Natal, Durban, South Africa; c School of Nursing, Faculty of Community and Health Sciences, University of Western Cape, Cape Town, South Africa; d Centre for quality Promotion and Assurance, Faculty of Health Sciences, Durban University of Technology, Durban, South Africa; e Executive Dean, Faculty of Health Sciences, Durban University of Technology, Ritson Campus, Durban, South Africa; f Executive Dean, School of Public Health, University of Venda, Thohoyandou, South Africa

**Keywords:** COVID-19, experiences, families, perceptions, people living with COVID-19

## Abstract

**Background:**

The incidence and prevalence of COVID-19 continues to escalate globally, with the consequence to quality of life, the economies of nations and various sectors of society. While there is substantial research on the impact and experiences of the COVID-19 pandemic, little remains known about the perceptions and lived experiences of families living with people diagnosed with COVID-19, particularly within the South African context.

**Purpose:**

To explore the perceptions and lived experiences of family members  living with people diagnosed with COVID-19 in South Africa.

**Methods:**

A descriptive phenomenological design was used. Data were collected from 15 participants who were family members of people diagnosed with COVID-19 in South Africa. Purposive snowball sampling was used to identify and recruit participants, and data were collected at community level in KwaZulu-Natal, Western Cape and Gauteng, South Africa. Individual in-depth interviews were used to collect the data, and an audio tape was used to record all interviews. Data were transcribed verbatim and analysed using a phenomenological data analysis processes. Ethical approval to conduct the study was obtained from the University of KwaZulu-Natal Research Ethics Committee—reference number: BREC00003228/2021.

**Results:**

Four super-ordinate themes emerged in relation to the perceptions and lived experiences of family members  living with people diagnosed with COVID-19 in South Africa. The superordinate themes were: (1) sources of information about COVID-19, (2) pandemic  perceptions and experiences, (3) impact of diagnosis and related burden and (4) aftermath of living with a family member diagnosed with COVID-19.

**Discussion and conclusion:**

Family members’ perceptions and lived experiences of COVID-19 are largely influenced by media, moreover, the impact of diagnosis has consequences for the physical, mental and emotional well-being of family members. Diagnosis disrupts family dynamics by depleting financial resources due to  the caregiver burden experienced. The findings thus imply that provision of psychosocial support is imperative for families living with persons diagnosed with COVID-19.

## Background

Coronavirus disease (COVID-19) is a pandemic, which was first diagnosed in Wuhan, China, in December 2019, and it became a public health emergency of concern across the (world Li et al. 2020). According to the World Health Organization (World Health Organization, 2022), approximately 608 848 852 confirmed COVID-19 cases and 6 507 002 COVID-19 related mortalities had been reported worldwide as of September 2022. In addition to this, it was reported that an estimated number of 12 613 484 608 vaccine doses were administered across the world. While the number of COVID-19 cases has been more prevalent in older people due to chronic illnesses, it has been highlighted that all ages are at risk of contracting the virus (Clark et al., 2020). In Africa, it was reported that there were approximately 8 804 381 confirmed cases of COVID-19 across 47 countries. In addition to the confirmed cases, approximately 173 398 COVID-19-related deaths were reported by September 2022 (WHO, 2022). According to the National Institute of Communicable Diseases (NICD) (2022), South Africa accounts for 4 016 157 confirmed cases of COVID-19 as of September 2022.

While the number of COVID-19 infections continues to escalate with many consequences, Lightfoot et al. (2021) have argued that the COVID-19 pandemic has greatly impacted individuals, communities and family members. Van Barneveld et al. (2020) and Kaye et al.(2021) further attest that the COVID-19 pandemic seriously impacted society, the economy, the medical system, and family members living with a person diagnosed with COVID-19. Beach et al. (2021) also postulated that the COVID-19 pandemic was likely to cause stress to the family of a person living with COVID-19. A recent study on family caregiving during the COVID-19 pandemic highlighted that family caregivers experienced a surge in their duties, responsibilities and burdens, which led to challenges which affected their quality of life, psychosocial aspects, and finances. Similarly, another study conducted by Lightfoot et al. (2021) revealed that caring for family members with COVID-19 was associated with decreased social and physical contact with patients living with COVID-19, which changed the care provision as other duties were not possible due to the restrictions with their loved ones. Another study by Sadler et al. (2021) revealed that family members of patients living with COVID-19 experienced challenges in assessing the quality of care they provided and were also unable to partake in medical appointments and communicate with their loved ones.

With an increasing incidence and prevalence of COVID-19 globally, the experiences and impact of the pandemic on lives and livelihoods continue to be reported globally from various perspectives, including healthcare workers, various categories of scholars, certain survivors and family members (Chersich et al., 2020; El Tantawi et al., 2022; Semo & Frissa, 2020). While research data on the experience of COVID-19 from the perspectives of family members exist, there is limited research on the lived experiences of family members living with persons diagnosed with COVID-19, especially in South Africa (Adebiyi et al., 2021; October et al., 2021). Attaining more knowledge about families’ perceptions and experiences about living with a person diagnosed with COVID-19 in South Africa may provide novel insights into the multiple effects of COVID-19 on quality of life and thus provides evidence to inform the development of holistic and comprehensive approaches to managing COVID-19 in this context. The aim of this study is thus to present findings on the perceptions and lived experiences of family members of people diagnosed with COVID-19 in South Africa. For this study, people diagnosed with COVID-19 refers to individuals who are considered as family by the participants (family members) in this study.

## Methods

### Study setting and design

The present study was conducted at the community level in different urban areas in South Africa. A qualitative approach, using a descriptive phenomenological design, was used to explore family members perceptions and lived experiences of living with people diagnosed with COVID-19 in South Africa. Descriptive phenomenology proposes that a phenomenon be described instead of explained or having its causal relationship investigated (Lee et al., 2014; Shorey & Ng, 2022). It examines and seeks to understand the subjective human experience regarding a phenomenon as it manifests in natural context (Matua & Van Der Wal, 2015; Sundler et al., 2019). Since there is paucity of research data on the perceptions and lived experience of family members living with persons diagnosed with COVID-19 in South Africa, descriptive phenomenology allowed for the illumination of poorly understood aspects of this human experience (Willis et al., 2016).

### Sampling and recruitment

Purposive snowball sampling was used to identify and recruit participants who reported having family members diagnosed with COVID-19 following the results of testing according to the South African health guidelines. Purposive snowball sampling was used in this study as it was deemed appropriate for gaining access to participants with specific characteristics, primarily those who were families that lived with people diagnosed with COVID-19 in South Africa. It also allowed for the effective recruitment of participants in line with the inclusion criteria of the study especially since data were collected at a community level.

The additional inclusion criteria for this study were: participants had to have been 18 years and older, living with the affected family member for a minimum of six months, status of COVID-19 infection was not a criterion for inclusion and exclusion hence participants that recruited were included those that had never been infected with COVID-19 and also those that were infected prior and during living with a family member diagnosed with COVID-19. Participants also had to be able to provide a verbal account of their experiences and consent to be audio-recorded.

To initiate the purposive snowball sampling approach, the researchers identified potential participants in their immediate sphere of contact by engaging with colleagues, neighbours, and members of the communities where the data were collected. Participants that were subsequently recruited in the initial stages of data collection were sampled purposively using a snowball approach through the networks of the researchers in the study. The sampling process occurred in the same period for all three provinces, as three out of five researchers were from the three different provinces selected for data collection. In this study, only three researchers collected data and conducted the initial preliminary analysis while the additional two independently analysed data as part of ensuring trustworthiness of the findings.

### Data collection

A pre-test was conducted with three participants before data collection to ensure clarity of the research questions. Contact numbers obtained during the identification of participants were used to set up appointments with participants at times convenient to them. Detailed information about the purpose of the study and the data collection process was provided to each participant. Furthermore, each interviewer explained that strict COVID-19 protection protocols would be followed during data collection. The three participants who formed part of the pre-test were not part of the main study, nor were these findings included in the main study’s results. There were subsequently no modifications required for the research questions. Data were collected in the respective communities within the three provinces identified for data collection. The data collection and analysis were done concurrently, and data collection ceased once data saturation had been reached. Three researchers collected data in the three provinces and analysed data independently during data collection. Data collection lasted for two weeks from 14 March 2022 to 29 March 2022. Following daily virtual engagement on emerging data, each researcher continued collecting data from one participant following data saturation to ensure no new information would emerge. An additional two researchers independently analysed data to ensure trustworthiness of emerging findings as initially analysed by the three researchers that collected data.

A semi-structured interview guide (see Appendix 1) was used to collect data using individual in-depth face-to-face interviews as participants in this study opted to respond as individuals on behalf of their families. Interviewers called participants to set up appointments at times convenient to the participants. The interview guide comprised of demographics section and one guiding question related to participants’ experiences of having a family member diagnosed with COVID-19. Probing was used to clarify questions, and interviews lasted between twenty-five and forty minutes. Interviews were conducted in English and in Isizulu in KwaZulu Natal. English and *IsiXhosa* languages were used to conduct interviews that were carried out in the Western Cape, and English and Sesotho were used to conduct interviews in Gauteng. The assistance of specialized language services was used for the transcription and translation of interviews conducted in IsiZulu, *IsiXhosa* and Sesotho, the University of KwaZulu-Natal’s language centre was used in this regard. All interviews were transcribed verbatim and kept in password-protected folders of the researcher’s laptops during data collection. The researchers had no prior experience of COVID-19 and in the field, the researchers made use of reflective journals. The use of reflective journaling was to document feelings, thoughts and perceptions during the interviews and analysis. This was also used to examine the researcher’s position on the issues raised and the emerging themes.

### Data analysis

Data analysis was guided by the phenomenological method of data analysis proposed by Colaizzi (1978) cited in (Shosha, 2012). Experienced researchers such as Chan et al. (2013) have successfully used the Colaizzi (1978) method of data analysis in their descriptive phenomenological studies. Data analysis was an iterative process that involved a step-wise approach as suggested by Colazzis steps of descriptive phenomenological data analysis. These steps included: (1) repeated reading of the transcripts to obtain a general sense of the whole content, (2) for each transcript, significant statements that pertained to the phenomenon under study were extracted and recorded, (3) meanings were then formulated from these significant statements, (4) the formulated meanings were sorted into categories, clusters of themes and themes, (5) the findings were integrated into an exhaustive description of the phenomenon under study, (6) The fundamental structure of the phenomenon was described, and (7) finally, member checking was done to validate the descriptive results.

The authors made a critical reflection of their own role and potential biases prior to analysing each transcript through the use of reflective journals to depict their own emotions and ideas about the research phenomenon. This facilitated an awareness of their own biases prior analysing each transcript and was discussed collectively before and after each transcript was analysed during and after data collection. The assistance of a fourth researcher to independently analyse to moderate discussion sessions and independently analyse and select the data to be included in the manuscript ensured that researchers’ biases were minimal, subsequently facilitating credibility of the research findings.

### Ethical considerations

The study was ethically approved by the University of KwaZulu-Natal Research Ethics Committee (Reference No. BEC00003228/2021). Gatekeeper permission was not necessary as data were collected in the community at participants’ homes. Informed consent was obtained from all participants verbally and in writing prior data collection. The researchers and acquaintances of the researchers provided information about the nature of the study during the sampling and recruitment phases. Once informed consent was obtained, participants were contacted telephonically to arrange for interviews. Furthermore, participants consented to conduct interviews at their homes if they were comfortable with the idea. All participants in this study consented to participate in this study during the first recruitment attempt, there were thus no participants that declined participation. Additionally, no remuneration was subsequently provided to participants as they incurred no direct costs because of the data collection process. The researchers additionally provided debriefing and counselling to all participants following data collection as this was a sensitive research subject. Additional follow-up was also done with participants between three to five days after data collection to determine if participants required additional psychological support. None of the participants required additional mental health support.

### Trustworthiness

Three researchers independently collected and analysed data and then consolidated findings which were presented. An additional two researcher who are experts in qualitative research methods also independently analysed data collected from the three provinces and compared the analysis with the results of the final version of the analysis to ensure credibility of the findings. Member checking was done to facilitate confirmability of the findings. To facilitate member checking, all participants were provided with copies of their interview transcripts as carried out in their respective native languages and participants were asked to review and provide feedback on their transcribed interviews. In addition, a preliminary analysis of individual transcripts was provided to allow them to review how their interviews were analysed. All participants agreed and confirmed that the transcripts and preliminary analysis were a reflection of their individual experiences.

The results are supported by verbatim quotations of participants’ statements to ensure dependability. Details of the design, sampling process, data collection, and data analysis procedures are provided to ensure transferability.

## Results

The sample comprised 15 participants who were both male and female and whose ages ranged between 24 and 66 years, from diverse ethnic groups with varying education levels. Pseudo-names are used to present the verbatim quotations of participants. See Table I for detailed demographic information on the participants. The study findings revealed four broad superordinate themes related to participants’ lived experiences related to COVID-19 in South Africa. These were: (1) sources of information about COVID-19, (2) pre-pandemic experiences, (3) pandemic experiences and (4) living with a family member diagnosed with COVID-19. There were subsequently twelve sub-ordinate themes arising collectively from all four superordinate themes. A summary of the emerging superordinate themes and their respective subordinate theme is outlined in Table II.

**Table 1.** Demographic profile of participants.

Participant	Pseudo name	age	Gender	Level of education	Household composition	Type of exposure to COVID-19	Comorbidities	Employment status
Participant1	Flora	44	Female	Highschool certificate	Family of four	Family related exposure to COVID-19	None	Unemployed
Participant2	Sara	66	Female	College diploma	Family of four	Family related exposure and confirmed positive case	Hypertensive and diabetic	Pensioner
Participant3	Jake	24	Male	College diploma	Family of six	Family related exposure and confirmed positive case	None	Self-employed
Participant4	Maggie	39	Female	Highschool certificate	Family of five	Family related exposure to COVID-19	None	Unemployed
Participant5	Philip	27	Male	College degree	Family of six	Family related exposure and confirmed positive case	None	Employed part-time
Participant6	Tammy	29	Female	College diploma	Family of eight	Family related exposure and confirmed positive case	None	Employed part-time
Participant7	Patricia	40	Female	Highschool certificate	Family of three	Family related exposure to COVID-19	None	Employed part-time
Participant8	Betty	44	Female	Highschool certificate	Family of Five	Family related exposure to COVID-19	Asthma	Unemployed
Participant9	Mandy	51	Female	University degree	Family of three	Family related exposure and confirmed COVID-19 positive case	Hypertensive	Self-employed
Participant10	Andrew	26	Male	University degree	Family of five	Family related exposure to COVID-19	None	Employed
Participant11	Sophia	48	Female	College diploma	Family of seven	Family related exposure and confirmed positive case	None	Employed part-time
Participant12	Susan	36	Female	College diploma	Family of four	Family related exposure and confirmed COVID-19 positive case	None	Employed part time
Participant13	Jerry	31	Male	College diploma	Family of six	Family related exposure and confirmed COVID-19 positive case	None	Employed
Participant14	Mark	26	Male	Highschool certificate	Family of three	Family related exposure to COVID-19	None	Employed part-time
Participant15	Peter	37	Male	College diploma	Family of four	Family related exposure and confirmed COVID-19 positive case	None	Employed

**Table 2.** Summary of themes and sub-themes.

Theme	Sub-theme(s)
1. Sources of information about COVID-19	1.1. Media coverage 1.2. General word of mouth and interpersonal relationships
2. Pandemic perceptions and experiences	2.1. Fear of infection and illness 2.2. Disinterest 2.3. Immediate concerns about COVID-19
3. Impact of diagnosis and related burden	3.1. Progression of disease and ill health 3.2. Multilevel stigma 3.3. Mental health challenges 3.4. Frustration with hospital protocols, procedures and COVID-19 regulations 3.5. Disruption of family dynamics
4. Aftermath of living with a family member diagnosed with COVID-19	4.1. Infection with COVID-19 4.2. Post COVI-19 condition

### Sources of information about COVID-19

This superordinate theme yielded two sub-ordinate themes concerning how participants received information about the COVID-19 pandemic. Two sub-ordinate themes emerged from this superordinate theme: media coverage, peer interactions, and general word of mouth.

### Media coverage

Participants in this study revealed that they became aware of COVID-19 through reports received through local news platforms such as television, radio, and electronic press. Participants also revealed that this information was also circulated on various social media platforms. The following excerpts from participants supported this:

on the TV, on the news. I heard about the Chinese people. We were watching them build new hospitals there, so we got really concerned about how people were just dying like, and the huge size of the hospitals they were building. (Flora, female, 44 years)

Mostly from the news, um, when the outbreak was announced, um, from [other cities] and yeah and basically how it was spreading through [countries] and yeah just kind of hearing about how it spread across the country… (Sara, female, 66 years)

Yeah, so, I first really saw it from the media and from the news. (Jake, male, 24 years)

### General word of mouth and interpersonal relationships

Participants also revealed that they had received information through third-party sources through peer information sharing using both formal and informal methods. The following statements supported this:

I think last year I heard it was going around in [a country], and then it must have been in January, most probably heard of it through word of mouth … (Maggie, female, 39 years)

I don’t know, I think it may be at the dinner table when I heard that there was a pandemic like COVID-19 (Philip, male, 27 years)

I heard people at work talking about this coronavirus that was an outbreak in [country](Tammy, female, 29)

### Pandemic perceptions and experiences

This superordinate theme revealed participants’ views and opinions regarding the COVID-19 pandemic concerning the nature of the disease, the potential health implications, and broader potential complications. This theme subsequently yielded three sub-ordinate themes: fear of infection and illness, disinterest, and immediate concerns about COVID-19.

### Fear of infection and illness

Participants reported that they were fearful about being infected with COVID-19 because they perceived that their health would deteriorate because of infection. Participants feared that infection would result in ill health and death like COVID-19 prevalence, incidence and mortality statistics reported by the media. The following statements supported this:

it is scary that it [inaudible] people that have a disease, that you don’t want to get it, and you are like, I don’t want to come into contact with those people … (Sara, Female, 66 years)

I don’t know, I was, like, with Ebola, I also had a bit of a gut feeling, and I was like, oh no, something could happen, so I was really afraid because people were really getting sick and dying” (Patricia, Female, 40 years)

Yeah, it just was scary, it felt like, um, something that would quickly spiral out of control if it wasn’t, yeah, if there weren’t any steps to mitigate against that.(Betty, Female, 44 years)

### Disinterest

Participants acknowledged that when they initially heard about COVID-19, they had no feelings about how the disease was or how it would progress because they felt that it would remain in China. They further admitted to underestimating the severity of the pandemic and the progress of infection and disease. Participants’ responses were as follows:

I didn’t think. I thought it was serious, but it did not seem to have that many deaths to people getting it … (Mandy, Female, 51 years)

So, it will, and the storm will go past, will pass us, like when we heard about the SARS and swine flu and all those things. So, it was like ok, it will be something that will pass and not be as severe or serious. (Andrew, Male, 26 years)

Well, in the beginning, we didn’t take it seriously because we thought it was going to be contained in China because we didn’t hear about it … (Tammy, Female, 29 years)

### Immediate concerns about COVID-19

Some participants expressed that they initially contemplated the potential impact of the COVID-19 pandemic in South Africa and were concerned because this is a low-resource setting. Furthermore, participants highlighted how their concern was aggravated because first-world countries could not manage the complications of infection because of the burden on the health system. The following statements supported this:

I also thought that we are not a first world country … . What is going to happen to us, you know, like the majority of people here? How is everybody going to be accommodated in hospitals, and you know, what will the healthcare situation be like, compared to western um, the west? (Sophia, Female, 48 years)

I saw what was happening in [other countries]. I thought that you had to do whatever you could not go to the hospital. (Flora, Female, 44 years)

I definitely felt like this feeling, like also a gut feeling, like almost apocalyptic, like this could spread everywhere and be catastrophic for us as a country. (Susan, Female, 36 years)

### Impact of diagnosis and related burden

Impact of diagnosis and related burden was a superordinate theme related to participants’ experiences of living with a family member diagnosed with COVID-19. This theme highlights the lived accounts of how the diagnosis impacted them individually and as a family unit. Subsequently, five sub-themes emerged from this superordinate theme: progression of disease and ill-health, multilevel stigma, mental health challenges, frustration with hospital protocols and procedures and disruption of family dynamics. Each of these is described below and is supported by the respective excerpts.

### Progression of disease and ill-health

Participants revealed how a family member’s health deteriorated because of the effects of infection and the general progression of the disease. Some participants also revealed how the symptoms commencing from testing to diagnosis and hospitalization increased in severity leading to complications such as lack of consciousness and death in certain instances. The following participant excerpts supported this:

As the days passed just, I mean, very out of breath, walking any sort of distance, whether it is not uphill or not downhill, it was just flat. Still she was like out of breath. (Maggie, female, 39 years)

For my aunt, it started with normal flu symptoms, and by the third day, she could not breathe, so we took her to the hospital. That when we found out she had COVID-19, she was eventually taken to ICU because her breathing was getting worse. (Andrew, Male, 26 years)

I remember before my dad was diagnosed, he was experiencing loss of taste. Then later that day, he could not smell [anything], and the next day his throat felt soar, he went for testing and was positive, and they quarantined my mother because she also tested after that, he was just lethargic as the days passed. (Susan, Female, 48 years)

### Multi-level stigma

Participants in this study revealed that they experienced stigma in many forms because of being associated with a close family member diagnosed with COVID-19. Some participants reported how work colleagues and other relatives stigmatized them during the quarantine period because of a confirmed COVID-19 diagnosis of a close family member. Participants also revealed how they experienced internal conflict in the form of self-blame when they were in contact with an immediate family member with COVID-19. Typically, this self-blame was because they felt that being in close contact with a family member with COVID-19 meant that they were potential sources of transmission and infection in areas such as the community, workplace and other distant family members during a social gathering. The following excerpts supported this:

No, they were—my daughter was cross. She was like get to your room and stay there. Do not come near us. (Flora, Female, 44 years)

it’s affecting now my family, and it’s affecting my colleagues and people around me, and yeah, one feels a bit, what do you call it, [inaudible], what is it in, like a leper [Laughter]. Don’t come near me, I’m a dangerous type of thing.(Patricia, Female, 40 Years)

I heard stories from a friend of how people at work did not want me to come back even after quarantine because they thought I was still infectious. (Maggie, Female, 39 years)

### Mental health challenges

Participants reported experiencing despair because of the limitations that arose due to being a close COVID-19 contact by virtue of a family member with a confirmed diagnosis. The quarantine and diagnosis of COVID-19 resulted in feelings of stress and depression for all family members. Moreover, participants also reported that they were anxious about the possible health outcomes related to infection and were directed at both the family member and them. The following excerpts supported this:

We had one evening with him that he was very out of breath, but he also has quite a bad anxiety, and I think it definitely as soon as you get more stressed about how out of breath you panic. (Patricia, Female, 40 years)

Like, coming back to my daughter, I think she kind of struggled with a little bit of depression because it was a little bit difficult for her to cope. (Flora, Female, 44 years)

I was anxious about what would happen to my mom and dad, especially because they were like 60, and we had seen how COVID-19 was in older people, so I thought about their health and things like death kept on creeping up.(Mark, Male, 26 years)

### Frustration with hospital protocols, procedures and COVID-19 regulations

Participants expressed dissatisfaction with several protocols and procedures that hospitals adhered to during the COVID-19 pandemic. Typically, participants revealed a dissatisfaction with the communication systems concerning the condition of a family member that had been admitted because of severe COVID-19-related illness. Other participants revealed that they received no communication about the condition of their hospitalized family member from the time of admission; information was only communicated during death. Participants further highlighted how the state of health services, resources and policies back then did not allow for any bonding with their families upon admission. However, this resulted in harsh separation, which was not followed up with communication or consideration of the psychological impact that the admission had on families at the time. The following excerpts supported this:

I was gravely frustrated obviously because it didn’t feel as though they took any information from one of next of kin or the family to update them or anything or at least so that when we do call, they know who is allowed to receive that information. (Sophia, Female, 48 years)

Also, just to add the hospital, my mom was in a private [hospital], but my aunt was in government, a government hospital. Um, when she got admitted, they never got details of her next of kin. So, we were never updated and didn’t know if anything was happening. They never called us to get permission for any procedures, um, because we [sic], I’m still not 100% sure what happened because they said that something happened with her trachea that caused her demise. (Jake, Male, 24 years)

When we were trying to plan for the funeral, um, she had a funeral policy, and we told them I could not come to the office because I had COVID-19. So, I needed them to assist me remotely, and they insisted we come in to do some documentation. And I’m like do you understand that I am a contact with someone who has COVID-19, I cannot come. (Tammy, Female, 29)

### Disruption of family dynamics

Participants revealed how the diagnosis of a family member affected many areas of their lives concerning social interactions, the health status of individual family members and dynamics of which had financial implications. Participants revealed that diagnosis of a family member resulted in isolation from social systems because of the compulsory quarantine, which impacted their ability to work. In certain instances, participants were booked off work, with subsequent lack of remuneration being provided during this period. The following excerpts from participants supported this:

The bad part of having COVID in the family was the separation from each other, I missed the interactions that we would have during dinner (Maggie, Female, 39 years)

I had to quarantine because I was a contact and where I work it’s no work no pay so that was a terrible time because the family had needs that I could not take care of. (Peter, Male, 37 years)

This period was difficult because one person got sick with COVID, but it became like everyone is sick because we are all close and living under one roof. (Jerry, Male, 31 years)

#### Aftermath of living with a family member diagnosed with COVID-19

This superordinate theme revealed participants’ events and occurrences related to COVID-19 infection when the pandemic was detected in South Africa. The findings revealed two sub-ordinate themes: infection with COVID-19 and post-COVID-19 conditions.

### Infection with COVID-19

Participants revealed how their family members contracted COVID-19 at some point. They also revealed how they had been in contact with family members who were diagnosed whilst living together in one household. Other participants revealed how they had been in a social gathering and later developed symptoms that necessitated testing for COVID-19, which subsequently led to being confirmed with this diagnosis. The following statements supported this:

I think a week later, and when I got home, she said she was feeling lethargic, um, she had lost her appetite. Yeah! So she was mostly in bed, and she wasn’t eating. So, she called the doctor and explained, and then she said it might be COVID-19, and she recommended a test but gave her some vitamins and some medication to treat the symptoms … the results later came back positive.(Mandy, Female, 51 years)

I actually got a bit of skrik [Panic] because then I thought, oh, I could be tired, and I could be a little bit fluey, but now that I couldn’t breathe suddenly, just with a flight of stairs, um then I got a bit of a skrik. So, as soon as we left, I went to get another test, and it came back positive. (Patricia, Female, 40 years)

She wasn’t feeling any symptoms for quite a long time. I think it must have been for like 5 five days or six days after I got it after I found out I was positive. (Jerry, Male, 31 years)

### Post COVID-19 condition

This sub-ordinate theme emerged from participants who reported being subsequently infected whilst living with a family member diagnosed with COVID-19. It describes participants’ accounts of the long-term health conditions secondary to COVID-19 infection. Participants generally revealed experiencing lower energy levels and long periods of unexplained fatigue, moments of breathlessness and hair loss. The following statement supported this:

um, they said yeah, I have damage in my lower lungs, and I have gone on a pump so, hopefully, that I can get back on exercise and things like that… (Tammy, Female, 29 years)

I experienced hair loss during and after months of getting COVID-19, and they say that is an indication from women that they say the trauma of getting to death, that women lose their hair. (Sophia, Female, 48 years)

I haven’t been able to do any exercise. If I do any exercise, I feel very lightheaded, I can’t breathe. My throat completely closes. It is like, I can go for a short walk, and still I feel very out of breath, but if I climb the flight of stairs, I am so out of breath compared with how I used to be. (Jake, Male, 24 years)

## Discussion

This study aimed to explore the perceptions and lived experiences of family members living with people diagnosed with COVID-19 in South Africa. The findings of this study suggest that the diagnosis of a family member with COVID-19 is also a major experience for people that live with them. Participants in this study revealed the multitude of challenges they experienced when a family member was diagnosed with COVID-19. The findings highlight the impact of life-threatening illnesses on the family unit and individual family members. This implies that psychosocial management of the individual together with their family, is important as they also experience trauma because of the diagnosis.

Based on the narration of participants’ experiences of living with a person diagnosed with COVID-19, perceptions about the pandemic were discovered about the nature of the pandemic. Participants harboured pandemic-related perceptions and experiences of COVID-19 in South Africa. The pandemic perceptions and experiences related to the information they received about COVID-19. Participants revealed that they received information from media, peer interactions and general word of mouth. This finding is indicative of the various sources of information that are available concerning COVID-19.

Moreover, this finding also highlights the role of media in influencing attitudes and perceptions about the pandemic. Research on the role of media during the pandemic has revealed that it has resulted in both negative and positive outcomes. Abbas et al. (2021) identified that while the excessive use of social media content during the global health crisis might have been useful for obtaining information, it also increased its toll on the health of society members by perpetuating distress and other psychological challenges. Concurring, Abd-Alrazaq et al. (2020) state that while social media provides a lucrative opportunity to facilitate health information dissemination to the public, it may also be destructive to public health efforts if not used appropriately. Other studies further allude to the role of mass media in circulating information that influences health behaviour, whether positive or negative (Ahmad & Murad, 2020; Bao et al., 2020). In this regard, effective interventions targeted at relevant authorities are necessary to address rumours and misinformation that result in negative behaviours associated with the misuse of mass media (Anwar et al., 2020; Liu et al., 2021; Tsoy et al., 2021).

The study also found that pandemic experiences and perceptions related to fear of illness, disinterest and immediate concerns about the pandemic also emerged. Generally, participants revealed experiences of fear and concern, whilst a minority expressed initial disinterest. Fear as an experience and perception related to the COVID-19 pandemic is a global phenomenon that has been widely cited in the literature and has been associated with the many mental health challenges that healthcare workers have reported and the general public (Cawcutt et al., 2020; Khattak et al., 2021; Urooj et al., 2020). Jørgensen et al. (2021) postulate that perception can be a culturally uniform determinant of preventive and avoidant forms of protective health behaviours. In the context of COVID-19-related fear, the facilitation of self-efficacy thus becomes integral to facilitating and maintaining protective behaviours (Harper et al., 2021; Wise et al., 2020). In the context of this study, the concerns expressed by participants may reflect participants lack of trust and faith in the South African health system and could be attributed to specific negative experiences related to access and quality of healthcare as cited in certain studies (Harris et al., 2011; Naidoo & Van Wyk, 2019). On the other hand, Quadros et al. (2021) found that fear was associated with individuals’ concern about getting infected or infecting their loved ones. The authors further illustrated that predominately women, young adults and information gathered through media were associated with fear of COVID-19. The finding of fear related to COVID-19 points to the need for designing interventions that reduce the potentially negative impact of this experience on the psychological well-being of individuals (Broche-Pérez et al., 2022).

Participants also narrated personal and family member-related experiences of being infected with COVID-19. Participants revealed the physiological symptoms that manifested, leading to testing and eventual diagnosis. These findings concur with the reported clinical manifestations of COVID-19 and reveal the contagious nature of the disease (Huang et al., 2020; Vetter et al., 2020; Zavascki & Falci, 2020). In contrast, Gao et al. (2021) reported evidence of increasing incidences of asymptomatic COVID-19 infections which pose a challenge to the prevention and control of the spread of infections from this type of patient. The collective findings highlight the need for rigorous contact tracing and epidemiological investigations to identify and manage the pandemic appropriately.

Participants further revealed their experience of post-COVID-19 conditions, which related to the pathological conditions experienced individually and by their family members, this was the aftermath of living with a family member diagnosed with COVID-19. According to Soriano et al. (2022) post-COVID-19 condition refers to the sustained sequelae of post-COVID-19 infection. While the epidemiology of post-COVID-19 conditions is relatively unclear, it is estimated that 10% to 15% of individuals infected with SARS-CoV-2 May experience this condition (Nalbandian et al., 2022). In this study, post-COVID-19 manifested as fatigue, hair loss and moments of dyspnoea. The results of a meta-analysis and review of the global prevalence of post-COVID-19 disease conducted by Chen et al. (2022) revealed that prevalence was substantial, with almost 200 million individuals experiencing long-term health-related consequences of COVID-19 infection. The clinical manifestations of post-COVID-19 condition were similar to those reported in the presented and in line with the case definition of the WHO (Soriano et al., 2021).

Post-COVID-19 infection is a significant finding in this study as it has implications for quality of life and healthcare in the South African context. Moreover, current evidence highlights the potential burden that this condition may have on the health system, especially since KwaZulu-Natal, South Africa, already has a quadruple burden of disease (Modjadji, 2021). The formulation of preventive interventions through education and screening approaches thus becomes integral from a health promotion perspective. Moreover, palliative and rehabilitative interventions must also be directed at individuals with post-COVID-19. These interventions must also reach family members living with persons diagnosed with COVID-19. According to Parkin et al. (2021), a comprehensive and integrated multidisciplinary approach to managing post-COVID-19 is adopted, encompassing the adoption of specialized multidisciplinary services, community therapy teams and the promotion of self-management of symptoms. Given the current disease burden on health care, adopting self-care and self-management could also be an element in the promotive, rehabilitative and palliative approach suggested to addressing post-COVID-19 in the South African context. Scordo et al. (2021) argue that patients and families who experience symptoms of post-COVID-19 condition also require targeted treatment and ongoing support.

Participants also revealed how they saw their family members’ health conditions deteriorating, leading to eventually poor health status and death in certain instances. This experience concurs with and reveals the traumatic nature of COVID-19 infection, as reported by previous studies on the effects of COVID-19 infection on individuals and families (Bonsaksen et al., 2021; Koçak et al., 2021; Vanderhout et al., 2020). This thus necessitates psychosocial interventions to address this emotional trauma through the provision of psychotherapy that it tailored to support the family unit and individual members of the family (Grover et al., 2020; James Riegler et al., 2020).

The experience of multilevel stigma (from community members, colleagues and other significant individuals of influence) was also reported by participants and related to being associated with a family member diagnosed with COVID-19. The stigma of COVID-19 is a widely reported phenomenon and is associated with the nature of the disease and clinical outcomes of infection (Bhanot et al., 2021; Samal, 2021; Schubert et al., 2021). The experience of stigma by association is a common experience that has been cited in other health conditions, such as mental illness (van der Sanden et al., 2013, 2015; 2016). Stigma, in general, denotes a mark of shame, fear or disgrace associated with a particular person, group of persons or circumstance (Link & Phelan, 2001). Stigma typically manifests as labelling, isolation, stereotyping and generally negative perceptions (Pryor & Bos, 2015). In the context of COVID-19 stigma can be understood as a social process that aims to exclude those who are perceived as a potential source of the disease and who may pose a threat to effective social life in a given society (Bhanot et al., 2021; Link & Cullen, 1983; Mikulincer et al., 2015). The issue of stigma continues to challenge many public health interventions that may be directed at both communicable and non-communicable diseases because it results in poor health-seeking behaviours which affect incidence and prevalence, thus compromising public health outcomes (Cook et al., 2014; Link & Phelan, 2006).

Participants also revealed experiences of mental health challenges of stress, depression and anxiety associated with the diagnosis of a family member with COVID-19 and related challenges such as stigma by association. This finding concurs with other studies conducted on the impact of COVID-19 on the family (Gayatri & Puspitasari, 2022; Tee et al., 2020). Research has also suggested that families often suffer mental health challenges such as stress, depression and anxiety when a family member is diagnosed with a life-threatening health condition (Darling et al., 2019; Hinojosa et al., 2012). These studies have thus advocated for providing care and support for families as they are often negatively affected during such times.

The frustration with hospital protocols, procedures and COVID-19 regulations was another experience that participants narrated. Regarding hospital protocols and procedures, participants expressed dissatisfaction with communication systems which were often fragmented regarding the clinical condition of their loved ones admitted due to COVID-19 illness. In certain instances, there was a lack of communication to the extent that their family members eventually demised without them being kept informed of the advancement of their conditions.

Research data on the global health response to the COVID-19 pandemic has revealed that most health systems were overwhelmed by the infection rates and resulting health complications. Previous studies reported on the issues of high patient-to-staff ratios that compromised service delivery and a shortage of crucial resources such as beds, ventilators, oxygen and even personal protective equipment (Kalateh Sadati et al., 2021; Liu et al., 2020; Nxumalo & Mchunu, 2021). This finding indicates the health system’s poor preparedness for the pandemic. It necessitates urgent policy and practice changes as it has implications for poor public health outcomes and may further perpetuate the mental health challenges experienced by individuals diagnosed with COVID-19 and their families. More importantly, there is a need for the development of a health system-and-family oriented communication strategy tailored to the unique needs of families and within the context of broader infectious and contagious diseases. This framework should consider the family as an integral part of the treatment and recovery process of the patient.

The disruption of family dynamics concerning health status, financial resources and social interactions was also experienced by participants in this study. This finding is possibly the very essence of the experience of people living with family members diagnosed with COVID-19. Concurring, Kalil et al. (2020) alluded that parents’ exposure to COVID-19 was associated with less positive parent-child interactions and resulted in several child behaviour challenges. On the other hand, Sun et al. (2021) found healthy sibling dynamics among children whose parents were affected by COVID-19 during the closure of schools. Nonetheless, another study on the impact of COVID-19 highlight the negative effects that the pandemic has had on families (Muldrew et al., 2022). This finding demonstrates that ill health affecting one family member has a negative impact on the entire family as a unit. This thus necessitates tailored interventions to address the unique needs of families as units and individual members that constitute the family unit (October et al., 2021).

## Implications and recommendations

The findings of this study suggest that living with a family member diagnosed with COVID-19, has multi-faceted implications affecting individual family members physical, mental and emotional well-being. This is related to the experiences of multi-levelled stigma, infection with COVID-19 and the aftermath of living with a person diagnosed with the disease. Moreover, the diagnosis of COVID-19 also results in disruptions to the family unit which have an impact on family relations, financial resources and the general well-being of the family unit. The study findings thus imply that holistic and comprehensive guidelines should be designed and implemented for families living with people diagnosed with COVID-19. These guidelines should be oriented to elements of promotive, preventive, curative and rehabilitative care with a strong emphasis on self-care and self-management particularly in relation to mental health challenges experienced and reduction of the risk and complications of infection with COVID-19.

The present study findings thus contribute to an awareness of how COVID-19 and future pandemics may have an impact of the livelihoods of families and family members living with persons affected. This thus necessitates multi-pronged policy and practice interventions related to the management of the specific pandemic.

## Strengths and limitations

While the design and overall aim of the study is not generalizability of findings, it provides an awareness and understanding of the perceptions and experiences of families living with persons diagnosed with COVID-19 in South Africa. The diversity of the sample characteristics and multiple data collection sites were a strength as this allowed for a wide range of experiences to be reported, especially since these provinces were reported to have the highest number of COVID-19 infections. Further research may be conducted to explore copying mechanisms employed by families living with persons diagnosed with COVID-19 and the potential impact of such copying mechanisms on the physical, mental and emotional well-being of families. This type of research may subsequently have implications for management guidelines orientated towards families affected by COVID-19.

Due to COVID-19 regulations at the time, certain interviews were conducted using online methods, which could have affected the richness and depth of the findings. Furthermore, certain participants included in this study were also infected with COVID-19, which could have influenced their experience of caring and living with the person diagnosed with COVID-19. Data collection also commenced with the initial recruitment of participants from the researcher’s own network and therefore sample bias may have occurred nonetheless the overall strengths of the study make these findings valuable.

## Conclusion

The findings of the present study suggest that families living with people diagnosed with COVID-19 experience several challenges related to the course infection and health outcomes of their family members. The study findings further suggest that the experience of living with a family member diagnosed with COVID-19 poses several health-related risks and related negative outcomes. Based on the findings of this study, comprehensive, tailored interventions are required to support people living with families and individual members diagnosed with COVID-19. Providing comprehensive and holistic psychosocial support services for families and individual family members is imperative as the diagnosis of COVID-19 also affects the family unit and individual members.

## Appendix 1

### INTERVIEW GUIDE FOR PARTICIPANTS

Dear Participant,

Thank you for your time and willingness to participate in this study.

This interview aims to explore perceptions and experiences regarding the COVID-19 pandemic as a family member living with an individual with a medical history of having COVID-19

Feel free to seek clarity or ask give more details when answering

If you need help, please indicate.

Thank You!

**INSTRUMENT 1**

INTERVIEW GUIDE FOR FAMILY MEMBERS AND PATIENTS/INDIVIDUALS WITH HISTORY OF COVID-19

**THE LIVED EXPERIENCES OF COVID-19 IN SOUTH AFRICA: A DESCRIPTIVE PHENOMENOLOGICAL CASE STUDY OF FAMILY MEMBERS**

**A: DEMOGRAPHICS**

**Table:**

**DEMOGRAPHICS**			
Age			
Gender	Male	Female	
Race/Ethnicity			
Level of education			
Household composition			
Type of exposure to COVID-19			
Comorbidities (Specify if any)			
Employment status			

**B: GUIDING INTERVIEW QUESTIONS**.

B1. How have you perceived and experienced the COVID-19 pandemic in South Africa?

B2. How have experienced living with a family member diagnosed with COVID-19 in South Africa?

Probe about: (1) fears, stigma, psychosocial needs, family related experience.

(2) experiences with community, health system, etc.

**Author Biosketches**

**Table:**

Author	Bio
1. Celenkosini Thembelenkosini Nxumalo	An academic development practitioner at the Durban University of Technology in South Africa and honorary lecturer and the University of KwaZulu-Natal. Presently holds a PhD in health sciences with a keen interest in HIV prevention, health systems and health policy research. Involved in teaching and learning at undergraduate and postgraduate level with an additional emphasis on enhancing the scholarship of teaching and learning
2. Lwandile Tokwe	Professional nurse and lecturer at the University of Western Cape, South Africa. Holds a Bachelor of and Master of Nursing degree respectively and a Postgraduate Diploma in Nursing education. He is also presently PhD candidate at Stellenbosch University, School of Nursing and Midwifery. Research interests are HIV & co-morbidities and how they are managed in rural PHC settings.
3. Silingene Ngcobo	Nurse academic currently at the School of Nursing and Public Health at the University of KwaZulu Natal, South Africa. She is involved in teaching and coordination of community-based education in the undergraduate nursing program at the university. Her research interest are HIV, mental health and women’s health.
4. Nkululeko Phalson Gam	A quality promotion officer in the faculty of health sciences at the Durban University of Technology. Responsible for quality assurance related to teaching, learning and assessment practices. He holds a PhD in health sciences with a research focus in quality health systems for radiography. His other research interests include advancement of the scholarship of teaching and learning within the context of a university of Technology.
5. Gugu Gladness Mchunu	Executive dean of the faculty of health sciences and executive member of the Durban University of Technology. She holds a PhD in Health Sciences (UKZN) and has more than 20 years’ experience in the academic sector. She has worked as an Occupational Health Nurse and as a Primary Care Nurse. She has served as an Executive Director of the Durban Chamber of commerce, a Senior Research Specialist at the Human Sciences Research Council (HSRC). Prof Mchunu has received a number of prestigious scholarships including The Ford Foundation International Scholarship Program and spent some time at the Johns Hopkins University, USA as a visiting scholar. She is the co-founding member for Action in Autism—an organization for people with Autism. Her research focus area is on Health Promotion in the Workplace.
6. Lufuno Makhado	An Associate Professor in the Department of Public Health. He has worked for over 11 years in the higher education sector. He specializes in Epidemiology and Biostatistics, HIV and AIDS research, Epilepsy, TB/HIV/NCDs integrated services, sexual and reproductive health, and trauma-related research and has published extensively in peer-reviewed and accredited journals.
